# The double loop mattress suture

**DOI:** 10.1111/wrr.12159

**Published:** 2014-04-02

**Authors:** John Biddlestone, Madan Samuel, Terry Creagh, Tariq Ahmad

**Affiliations:** 1 CRUK Clinical Research Fellow, Centre for Gene Regulation and Expression, College of Life Sciences, University of DundeeDundee, United Kingdom; 2 Honorary Speciality Registrar, Department for Plastic and Reconstructive Surgery, Ninewells HospitalDundee, United Kingdom; 3 Consultant Plastic and Reconstructive Surgeon, Addenbrooke's HospitalCambridge, United Kingdom; 4 Consultant Paediatric Surgeon, Mediclinic City HospitalDubai Healthcare City, Dubai; 5 Consultant Plastic and Reconstructive Surgeon, Christchurch HospitalCanterbury, New Zealand

## Abstract

An interrupted stitch type with favorable tissue characteristics will reduce local wound complications. We describe a novel high-strength, low-tension repair for the interrupted closure of skin, cartilage, and muscle, the double loop mattress stitch, and compare it experimentally with other interrupted closure methods. The performance of the double loop mattress technique in porcine cartilage and skeletal muscle is compared with the simple, mattress, and loop mattress interrupted sutures in both a novel porcine loading chamber and mechanical model. Wound apposition is assessed by electron microscopy. The performance of the double loop mattress in vivo was confirmed using a series of 805 pediatric laparotomies/laparoscopies. The double loop mattress suture is 3.5 times stronger than the loop mattress in muscle and 1.6 times stronger in cartilage (*p* ≤ 0.001). Additionally, the double loop mattress reduces tissue tension by 66% compared with just 53% for the loop mattress (*p* ≤ 0.001). Wound gapping is equal, and wound eversion appears significantly improved (*p* ≤ 0.001) compared with the loop mattress in vitro. In vivo, the double loop mattress performs as well as the loop mattress and significantly better than the mattress stitch in assessments of wound eversion and dehiscence. There were no episodes of stitch extrusion in our series of patients. The mechanical advantage of its intrinsic pulley arrangement gives the double loop mattress its favorable properties. Wound dehiscence is reduced because this stitch type is stronger and exerts less tension on the tissue than the mattress stitch. We advocate the use of this novel stitch wherever a high-strength, low-tension repair is required. These properties will enhance wound repair, and its application will be useful to surgeons of all disciplines.

The holding capacity of an interrupted suture depends on the strength of the suture material, tissue strength, type of knot, distance from the cut edges, and importantly, the suture technique employed.^1^ A combination of the fixed nature of tissue strength and recent advances in the strength of suture materials has shifted the focus of interrupted wound closure to that of suture technique.

The goal of wound closure is a satisfactory wound apposition, a procedure that necessitates the application of a suture pull (effort) equal to that of tissue recoil (load); however, it is the distribution of this effort in the wound edge that is suture conformation specific. Poor distribution of the suture pull (effort) can lead to microvascular compromise, inflammation, wound pain, and increases in the local complications of wound closure, such as dehiscence, necrosis, infection, and subsuture scarring.^2^,^3^ The ideal stitch would be strong and exert minimal tissue tension; however, this combination can be difficult to achieve with satisfactory wound apposition.

The conventional loop mattress suture was described by Gault and colleagues in 1987^4^ as a simple modification of the traditional mattress stitch.^5^ The loop mattress solved some of the problems of suture pull (effort) balancing by reducing suture tension and increasing suture holding capacity while maintaining good dermal apposition on distraction with straightforward removal. Since its inception, the loop mattress stitch has remained the gold standard for high-strength, low-tension interrupted wound repair. Minor modifications of the original conformation have since been described,^6^,^7^ but the mechanical advantage has remained constant. The intrinsic strength of this stitch lies in the fact that the loop acts as a pulley to distribute the force evenly throughout the suture strand, which crosses the wound four times.

Here, we describe a modification of the loop mattress suture that represents a novel and significant advance in interrupted wound closure: the double loop mattress stitch.

## Double loop mattress technique

**Figure 1:**
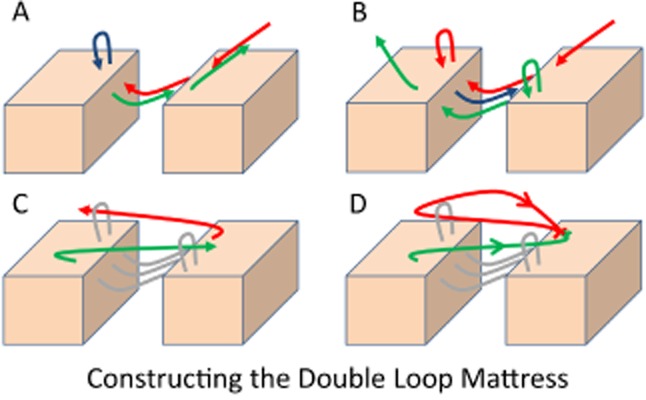
Trailing end is red and leading end is green. Arrows denote direction of suture. Loops are formed by combining two horizontal mattress stitches (A and B). The leading and trailing strands cross the wound again to pass through their respective loops before being securely tied (C and D). A video of the double loop mattress construction is available online to enhance this figure.

The double loop mattress stitch is constructed as illustrated in Figure 1. Supporting Information Video S1 of the surgical technique is available with the online version of this article. The first loop is generated by passing the leading strand through the near and far, then far and near wound edges (similar to the loop mattress stitch construction—Figure 1A). The second loop is made by passing the leading strand from near to far wound edges (Figure 1B). The stitch is tied after passing the trailing strand through the first loop and the leading strand through the second loop (Figure 1C and D). In the double loop conformation, the suture strand crosses the wound six times. The second loop creates a second pulley which provides an additional mechanical advantage that increases the strength and reduces the tension across the wound when compared with the loop mattress stitch.

To explore the properties of this suture conformation, we loaded the double loop mattress and the three types of established interrupted stitch (simple, mattress, and loop mattress) with a progressively increasing distracting force. To draw an accurate comparison, we recorded the maximum force achievable for each suture confirmation in different tissue types and the tissue deformation that occurred prior to achieving the maximum force.

## MATERIALS AND METHODS

### Sample procurement and preparation

UK domestic pig, full-thickness dermis, ear, tibialis anterior tendon, and digastric skeletal muscle were harvested daily, five minutes postmortem (Dalehead Foods Ltd. Linton, Cambridgeshire, United Kingdom). All tissues used in this study were procured from animals that had been humanely treated in accordance with National Institutes of Health guidelines. Animal procedures were reviewed and approved by the University of Cambridge animal care and use committee. Samples were dissected en bloc and fast-chilled to 4 °C in pregassed (95% O_2_, 5% CO_2_) Krebs–Hensellite Ringer (KHR), pH 7.4 (118 mMol NaCl, 4.7 mMol KCl, 0.94 mMol CaCl_2_.2H_2_O, 25 mMol NaHCO_3_, 1.2 mMol MgSO_4_, 1.2 mMol KH_2_PO_4_, 5.6 mMol Glucose) (Reagents courtesy of University of Cambridge Department of Pharmacology). KHR is a physiological ringer solution that meets the metabolic requirements of porcine skin, muscle, and cartilage and prevents sample degradation. All further sample preparation was performed in KHR at 4 °C.

### Muscle

The anterior belly of digastric was divided close to its insertion in the mandibular digastric fossa, and the maximum amount of intermediate tendon was preserved. The dissected muscle was joined to a section of tibialis anterior tendon using the suture conformation under test. Muscle viability was confirmed by brief stimulation with acetylcholine prior to sample mounting.

### Cartilage

Uniform elastic cartilage sheets with intact perichondrium were harvested from the en bloc ear specimens by sharp dissection. Twenty-five millimeter square samples were prepared using a tissue punch and symmetrically divided. Newly divided segments were then rejoined using the suture conformation under test.

### Skin

Samples of porcine facial dermis were uniformly wounded with a 5-mm elliptical tissue punch then immediately reapposed using the standard suture material and the suture conformation under test. Reapposed samples were sent for an assessment of apposition by scanning electron microscopy (SEM).

### Experimental chamber

**Figure 2:**
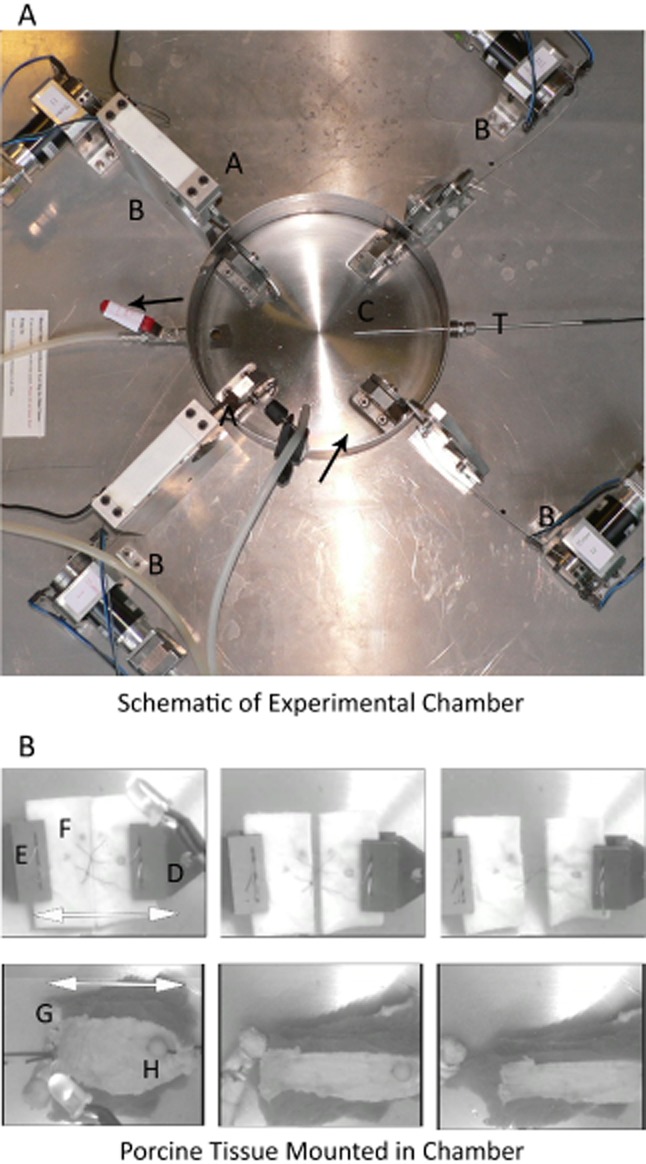
(A) In vitro experimental chamber: The chamber (*C*) is constructed from stainless steel and is mounted on a vibration-isolating platform. Two Load cells are positioned on perpendicular axes (*A*). Force is generated by activation of the stepper motors (*B*). Pregassed and warmed Krebs–Henseleit ringer is pumped into the chamber from a water bath (not shown) and circulates in the direction of the black arrows. The temperature of the chamber is constantly monitored by the retractable probe (*T*).(B) Mounted tissue. Upper three panels left to right: Cartilage samples are mounted in the chamber using steel clamps (*D*) and stress pins (*E*) in the orientation of axis I (white arrow). Reference points are marked onto the cartilage to allow displacement to be measured (*F*). The synchronizing light emitting diode is removed once triggered. Lower three panels left to right: Muscle samples are mounted in the chamber in a similar manner to cartilage samples. The joined tendon is in view (*G*). A sizing pin of known diameter is used as a reference point (*H*).

A bespoke physiological experimental chamber was constructed from stainless steel (Figure 2A) (University of Cambridge Department of Materials Science and Metallurgy, Cambridge, United Kingdom). The chamber was temperature controlled (Labfacility, Sheffield, United Kingdom; XF-315-FAR) and designed to maintain samples in gassed KHR (95% O_2_, 5% CO_2_) at 37 °C throughout the test period. Stepper motors were used to apply a uniformly incremental distracting force (0.5 mm/s) in to all samples. Multiple load cells were positioned to record real-time measurements of force (Tedea-Huntleigh Model 1022; InterTechnology Inc. Toronto, Canada;). The charge coupled device (CCD) recorded images of sample displacement that were synchronized with the load cell measurements using a light emitting diode (LED) trigger. All components were controlled, and data acquisition was performed using an implementation of LabVIEW software (National Instruments, Berkshire, United Kingdom).

Complete muscle preparations were mounted using stress pins parallel to the direction of the suture. A sizing pin was sited in each muscle sample proximally to calibrate the image measurements. Sample images of mounted muscle and cartilage preparations are shown in Figure 2B.

Complete cartilage preparations were mounted using steel clamps and stress pins parallel to the direction of the suture and marked at either side of the iatrogenic wound to measure displacement.

### Mechanical model

The mechanical advantage of each suture conformation was measured using a bespoke mechanical model (University of Cambridge Department of Materials Science and Metallurgy), wire cut from mild steel (Figure 4A). The sliding segment and loop facets were seated in a bed of virgin 100% polytetrafluroethylene to allow for frictionless movement (Engineering and Design Plastics Ltd., Cambridge, United Kingdom). For each stitch conformation, the suture material was arranged across the relevant, low-friction steel pulleys and resting state marked. A 2N load was applied across the sliding segment using a hanging weight system to simulate wound recoil (load). A constant force was applied to simulate the effort of closure, and the running tension to balance the load was measured using a load ring. Apposition was deemed complete when both effort and load were balanced and the sliding segment was returned to the resting position.

### Suture material

5-0 Prolene polypropylene monofilament (PC-3, 8635G; Ethicon, Johnson and Johnson Medical Limited, Livingston, Scotland) was used in both the porcine and mechanical model experiments. Where required, all stitches were secured using a square throw reef knot with two locking loops. Following completion of each experiment, the remaining fractured sutures were retained for analysis by light microscopy to rule out material defects or knot fracture.

### Light microscopy

Light microscopy with phase contrast was performed using the ×5 objective of a Zeiss AxioLab.A1 widefield microscope (Carl Zeiss Microscopy GmbH, Jena, Germany) to exclude material defects in the suture material following fracture.

### Electron microscopy

Two skin samples of each suture type were fixed and sent for analysis of tissue distortion and apposition by SEM. A copper splint was glued over the apposed wound to prevent skin curling or tissue distortion during fixation. Samples were fixed for a minimum of 48 hours in 4% gluteraldehyde and phosphate buffer, the copper splint was removed, and the samples were postfixed in osmium tetroxide, dehydrated in ethanol, and critical point dried. Samples were sputter-coated with gold prior to SEM examination. Horizontal and vertical wound edge apposition was measured at five nonconsecutive points within the limits of the stitch boundaries for each stitch type using Image J software (NIH, Bethesda, MD) from anteroposterior (AP) and oblique (O) SEM images, respectively (Figure 5A).

### In vitro data analysis

Raw load cell data were converted to force and synchronized with CCD images as previously described. Data analysis was performed using SigmaPlot v12.0 (Systat Software Inc. London, United Kingdom) and Image J software. Interstitch comparison is by one-way ANOVA with Holm-Sidak pairwise comparison. Displacement data was extracted from the CCD images in Image J. The modulus of elasticity (Young's modulus) was calculated by linear regression of the stress/strain curves for each stitch type. The modulus of resilience was calculated by measuring the area under the elastic portion of the stress-strain curve using a macro. Data are presented as category mean ± standard error of the mean unless otherwise stated.

### In vivo case series

We conducted a nonrandomized prospective linear observational study, which was case controlled. In the past 5 years (January 2008 to December 2012), 805 laparotomies/laparoscopies were performed on 772 children for wide variety of conditions that included tumor resection, bowel resection for necrotizing enterocolitis, bowel atresias, intestinal duplication, colectomy, closure of colostomy/enterostomy, pyloric stenosis, and resection of Meckel's diverticulum, hemangiomatous bowel, intestinal perforation, gall bladder pathology, splenectomy, intussusceptions, and appendicitis. The data were collated prospectively on a pediatric surgery database. The variables collated for the purpose of this study were suture type, suture size, type of wound closure, and outcomes. The data were collated and analyzed on Microsoft Excel (Microsoft Corp, Redmond, WA). The variables that were standard in all laparotomy/laparoscopy surgery were preoperative skin preparation, suture material, and usage of disposable drapes and gowns. Chloraprep (2% chlorhexidine gluconate and 70% isopropyl alcohol; CareFusion, San Diego, CA) was used for skin preparation preoperatively in all patients. Sutures used were PDS II (Polydioxanone) (Ethicon Inc., Johnson & Johnson Company, Norderstedt, Germany) for abdominal closure and subcutaneous fat. Vicryl (Polyglactin 910) (Ethicon Inc., Johnson & Johnson Company) was utilized for subcuticular skin approximation.

Of the 805 wounds, 539 were closed by interrupted loop mattress, 199 by mattress, and 67 by double loop mattress stitches. In 539 patients, the peritoneum was closed by a continuous running suture and the muscle layer en masse using interrupted loop mattress. In 199, the peritoneum was closed by a continuous running suture, and muscle was approximated by interrupted mattress suture. In 67, the peritoneum and the muscle layer were closed en masse using the interrupted double loop mattress. In all patients, the subcutaneous layer was closed by interrupted simple stitches. Skin was approximated by continuous subcuticular suture.

The outcome parameters assessed were wound edge eversion, suture extrusion, superficial wound infection, and wound dehiscence. Mean and proportions were compared by standard Student's *t* test and confidence intervals (CIs). Mann–Whitney *U* test was used for comparison of nonparametric data. Consent was obtained from all parents for enrolling their children in the study. Local ethics committee approval was obtained.

### Dressing clinic assessment

Five experienced plastic surgery nurses in a single unit (TC) who had removed at least 20 double loop mattress sutures in patients who had undergone keystone flap soft tissue repair were asked to complete a modified Likert scale (1 = poor; 5 = good) to compare wound healing and suture removal with the loop mattress suture. Data are presented as mean ± standard error. Interstitch comparison is by one-way ANOVA.

## RESULTS

### Experimental model: cartilage

**Figure 3:**
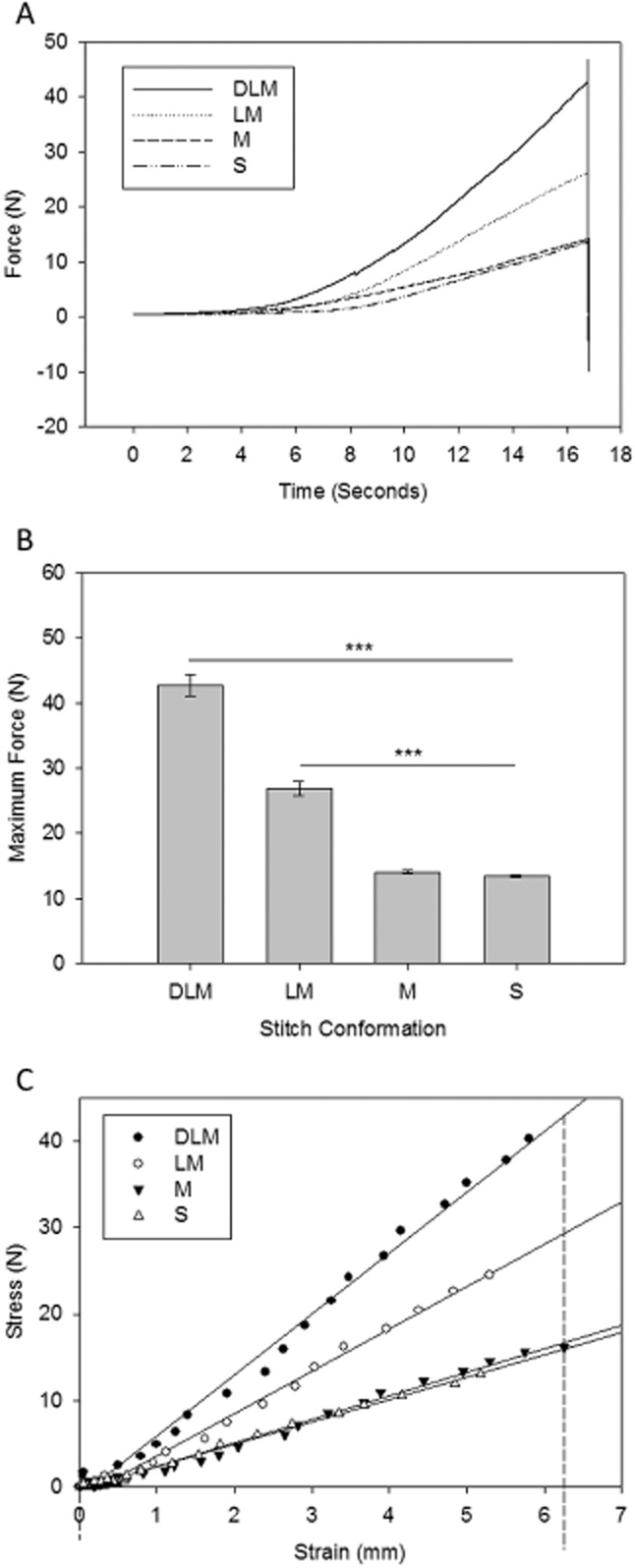
(A) Sample data from cartilage experiments to show the variation in force generated between the stitch types. Double loop mattress (DLM), loop mattress (LM), mattress (M), and simple (S). (B) Bar chart to show the maximum mean force generated following the application of a distracting force to each stitch type in cartilage. (C) Stress: strain scatter plot sample data for each stitch type in cartilage. The tissue displacement is measured on the images captured as the sample is progressively stressed. Linear regression lines are overlaid for each stitch type. The slope of the regression gives the modulus of elasticity, and the areas under the curves (dotted drop lines) are equal to the modulus of resilience.

A near-uniform sheet of type II cartilage provides support for the porcine ear anatomically;^8^ this cartilaginous sheet is easily accessible and of a high tensile strength. In this series of experiments, squares of cartilage were divided then rejoined using the stitch under test (Figure 2B). Rejoined squares of cartilage were then subjected to a distracting force to the point of suture fracture. Measurements of the overall force and the displacement of the cartilage were made in real time. Graphs of force vs. time were produced from these data; a sample is shown in Figure 3A). Twenty repeats were performed for each stitch conformation under test. The mean force at suture fracture in cartilage was 13.46 N (±0.21) for the simple, 14.04 N (±0.28) for the mattress, 26.85 N (±1.05) for the loop mattress, and 42.71 N (±1.60) for the double loop mattress (Figure 3B). The double loop mattress stitch is significantly stronger than the loop mattress (1.6×), mattress (3.0×), and simple (3.2×) sutures in cartilage (*p* ≤ 0.001). The loop mattress was also significantly stronger than the mattress or simple stitches (*p* ≤ 0.001). There was no statistically significant difference between force at suture fracture for the simple and mattress sutures (*p* = 0.674).

**Figure 4:**
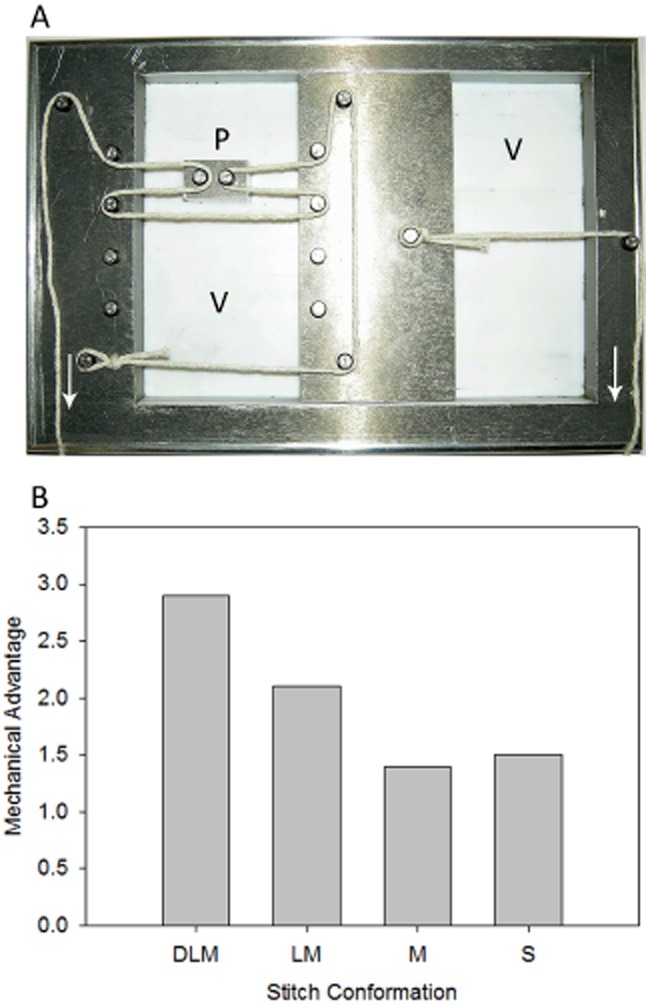
(A) Mechanical model: This bespoke rig was wire cut from mild steel and seated in a bed of virgin polytetrafluroethylene (PTFE) (V) to operate a frictionless array of pulleys (P) for any suture confirmation under test. A fixed load was applied to stimulate wound recoil (right side). The effort required to overcome this was measured using a load ring attached to the opposite end of the rig (left side). String in loop mattress conformation is used for illustrative purposes only. (B) Bar chart to show the actual mechanical advantage of each stitch type. The double loop mattress (DLM) provides an advantage of 2.9 which compares favorably with the other stitch types tested.

**Figure 5:**
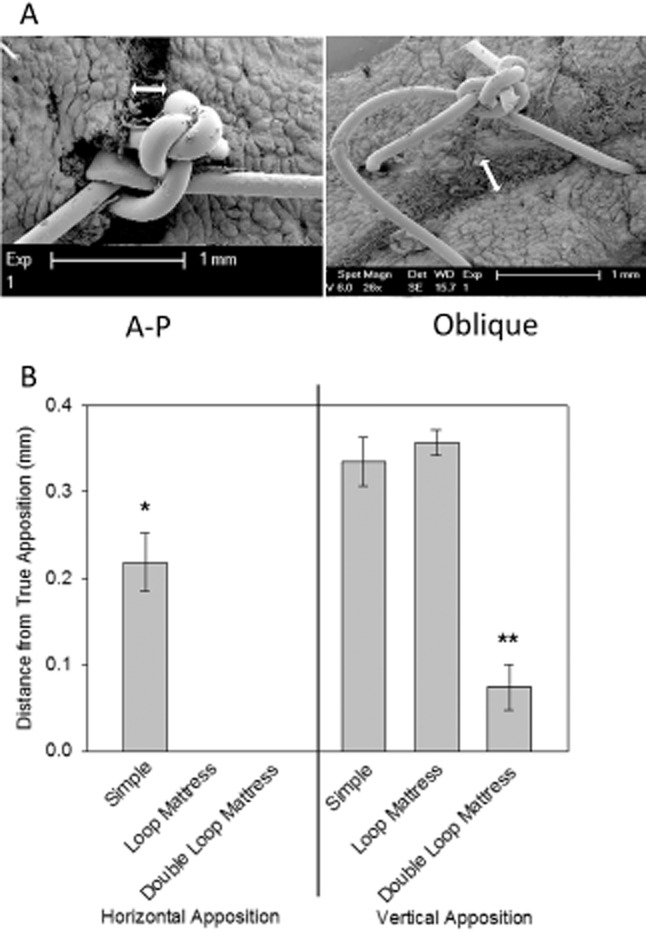
(A) Sample scanning electron microscope images. Left: anteroposterior (A-P) image used to assess horizontal wound apposition (white arrow). Right: oblique image used to assess vertical wound apposition (white arrow). (B) Bar chart to show the outcomes of the assessment of vertical and horizontal apposition by electron microscopy. Horizontal apposition is analogous to wound gapping, whereas vertical apposition is analogous to wound eversion.

To test the effect of suture conformation on tissue elasticity, the tissue displacement was extracted from the CCD images and plotted against force. The slope of the linear portion of the resulting stress–strain curve gives the modulus of elasticity (Young's modulus) for each stitch conformation: the higher the modulus of elasticity, the stiffer the material under test. Examples of this type of stress/strain graph are shown in Figure 3C. The area under the linear portion of the stress–strain curve gives the modulus of resilience, a measure of the ability of the material under test to absorb energy without creating a permanent distortion. These measures are both useful because they provide evidence of the change in tissue characteristics that are conferred by a change in suture conformation.

Five repeats were performed for each stitch conformation under test. The mean modulus of elasticity in cartilage was 2.323 N/mm^2^ (±0.205) for the simple, 1.939 N/mm^2^ (±0.160) for the mattress, 4.639 N/mm^2^ (±0.215) for the loop mattress, and 7.302 N/mm^2^ (±0.312) for the double loop mattress. The double loop mattress stitch conformation is seen to make the cartilage significantly stiffer than the loop mattress (1.5×), mattress (3.6×), and simple (3.0×) sutures (*p* ≤ 0.001). The loop mattress stitch conformation also made the cartilage significantly stiffer than the mattress or simple stitch conformations (*p* ≤ 0.001). There was no statistically significant difference in cartilage stiffness between the simple and mattress suture conformations (*p* = 0.255).

After calculation of the area under the stress–strain curve, we found that the mean modulus of resilience in cartilage was 36.647 N/mm^2^ (±4.160) for the simple, 47.170 N/mm^2^ (±8.886) for the mattress, 49.910 N/mm^2^ (±4.563) for the loop mattress, and 105.913 N/mm^2^ (±11.240) for the double loop mattress. These data show that the double loop mattress stitch conformation allows the cartilage to absorb significantly more energy than the loop mattress, mattress, and simple sutures (*p* ≤ 0.001). Interestingly, there was no statistically significant difference in the ability of the cartilage to absorb energy between the three remaining suture conformations (*p* ≥ 0.05).

### Experimental model: muscle

Porcine skeletal muscle has been shown to be almost identical to human skeletal muscle in function, metabolic demand, and pathology.^9^=^11^ The ordered, unidirectional architecture of skeletal muscle makes its repair difficult because of the high incidence of suture extrusion.^12^ In this series of experiments, the anterior belly of digastric was divided and joined to a section of tibialis anterior tendon using the stitch under test (Figure 2B). The samples were mounted in the experimental chamber (Figure 2A) and then subjected to a distracting force to the point of suture fracture or extrusion. Measurements of the force were made in real time. Fifteen repeats were performed for each stitch conformation under test. The mean force at suture fracture or extrusion was 2.19 N (±0.14) for the simple, 2.58 N (±0.12) for the mattress, 4.26 N (±0.23) for the loop mattress, and 15.04 N (±0.82) for the double loop mattress. In porcine muscle, the double loop mattress stitch is significantly stronger than the loop mattress (3.5×), mattress (5.8×), and simple (6.9×) sutures (*p* ≤ 0.001). The loop mattress was also significantly stronger than the mattress (*p* = 0.017) or simple stitches (*p* = 0.004). There was no statistically significant difference between force at suture fracture for the simple and mattress sutures (*p* = 0.523).

### Mechanical model

To examine the mechanical advantage of each suture conformation, a bespoke mechanical rig was constructed (Figure 4A). Each suture conformation was tested against a fixed load of 2N to simulate wound recoil. A load ring was used to measure the effort required to appose the load according to stitch type. Five repeats were performed for each stitch conformation under test. The mean effort required to appose the load was 1.332 N (±0.005) for the simple, 1.428 N (±0.009) for the mattress, 0.948 N (±0.005) for the loop mattress, and 0.685 N (±0.007) for the double loop mattress. The actual mechanical advantage (AMA) is calculated as the load (2N) divided by the effort required to appose the wound. The mechanical advantage was 1.5 for the simple, 1.4 for the mattress, 2.1 for the loop mattress, and 2.9 for the double loop mattress (Figure 4B). The percentage effort required to move a fixed load is 66.6% (±0.2) for the simple, 71.4% (±0.4) for the mattress, 47.4% (±0.3) for the loop mattress, and 34.2% (±0.4) for the double loop mattress. These data show that the double loop mattress stitch confers a significant mechanical advantage over the loop mattress, mattress, and simple conformations (*p* ≤ 0.001). A significant difference was detected between all groups with the loop mattress showing mechanical advantage over the mattress and simple stitches (*p* ≤ 0.001), and the simple stitch showing mechanical advantage over the mattress stitch (*p* ≤ 0.001).

### Light microscopy

Fractured sutures were retained for examination by light microscopy following sample testing in the experimental chamber. A total of 97 fractured sutures were retrieved. Failure of recovery occurred in 17 cases as the result of suture loss to the irrigation system, and 46 sutures were extruded in the muscle experiments. 10 fractured sutures for each stitch conformation were selected at random for examination by light microscopy. Suture failure due to fracture at the knot itself occurred once for each of the simple and mattress groups, respectively. All other sutures had failed at sites distant from mechanical manipulation. No statistically significant difference was seen between groups (*p* ≥ 0.05).

### Electron microscopy

Two samples of each stitch conformation were fixed and sent for SEM to accurately assess tissue apposition. Images were taken from both AP and O angles to give the best possible three-dimensional assessment of apposition. Examples are shown in Figure 5A. The mattress suture samples were damaged during fixation and were excluded from further assessment. Horizontal and vertical wound edge apposition was measured at five nonconsecutive points within the limits of the stitch boundaries for each stitch type from AP and O images. There was a significant reduction in horizontal apposition (wound gapping) for the double loop mattress and loop mattress conformations compared with the simple conformation (*p* < 0.001). No significant difference in horizontal apposition was noted between the loop mattress and double loop mattress stitches. There was a significant improvement in vertical apposition (wound eversion) for the mean vertical displacement of the double loop mattress (0.07 mm ± 0.01) compared with both the loop mattress (0.36 mm ± 0.01) and simple stitch (0.33 mm ± 0.01) conformations (*p* < 0.001). No significant difference in vertical apposition was noted between the simple and loop mattress stitches (Figure 5B).

### In vivo case series

In the past 5 years, 352 supra or infraumbilical incisions were performed for minilaparotomies or for laparoscopic surgery and 453 laparotomies using either an upper or lower quadrant transverse muscle cutting abdominal incision. The age distribution was neonates (29–42 weeks), *n* = 131; infants (43 weeks to 12 months), *n* = 103; 13–24 months, *n* = 155; 25 months to 5 years, *n* = 141; and 5.1–15 years, *n* = 275. There were 532 (66%) clean and 273 (34%) contaminated coelomic cavities at operation. Irrespective of type of closure, there was no suture extrusion in any of the 805 closures. Good wound edge eversion was achieved in single and double loop mattress; 606 (75%) in comparison edges were approximated without eversion in 199 (25%) of mattress closures (*p* = 0.012; CI 0.001–0.011).

The overall wound infection rate was 1.6% (13/805). In six (46%) neonates with contaminated coelomic cavities because of intestinal perforation from NEC, two of six had mattress closure. Both developed wound infection and subsequent wound dehiscence, which required secondary closure using loop mattress sutures. Following secondary closure, there were no further infections or dehiscence. In comparison, the other 4/6 neonates had wound infection of the dermal and subcutaneous plane that did not extend to the muscle layer. These four of six had undergone loop mattress closure of the muscular layer (*p* = 0.0001; CI 0.00121–0.01422). The superficial wound infection was treated conservatively. In the other 7/13 (0.9%) laparotomies, five (39%) were contaminated, and two (15%) were clean. All had superficial wound infection and were treated conservatively. The mode of closure was 6/13 (46%) mattress and 1/13 (8%) loop mattress.

Incisional hernia occurred in 0.5% (4/805). Three were umbilical incisions, and one transverse incision following colostomy closure. All had undergone simple mattress closures. In comparison, there were no incisional hernias following loop mattress (*p* = 0.0001) and double loop mattress (*p* = 0.0001) closures. There were no complications in the cohort that had undergone double loop mattress closure. All these patients (67/805: 8.3%) had clean laparotomy for tumor resection. In 33 who underwent secondary laparotomy, the mode of closure was loop mattress (14/33: 42%) and double loop mattress (19/33: 58%). There was no wound dehiscence.

We also examined the early outcomes and ease of removal of this stitch type compared with the loop mattress in the repair of soft tissue defects using the keystone flap. We asked experienced plastic surgery nurses to rate the double loop mattress for ease of removal, local wound healing, and overall impression compared with the loop mattress stitch. The double loop mattress suture was comparable with the loop mattress in all areas assessed. The double loop mattress suture was as easy to remove as the loop mattress (score = 2.8 ± 0.37, *p* = 0.608); there were no significant differences in wound healing between stitches (score = 2.8 ± 0.20, *p* = 0.347), and the nurses rated the double loop mattress as comparable overall to the loop mattress stitch (score = 2.8 ± 0.49, *p* = 0.694).

## DISCUSSION

We have described the use of novel porcine ex vivo and mechanical rigs to examine the loading properties of three established interrupted sutures. In agreement with the findings of Gault and colleagues,^4^ we confirmed the efficacy of the loop mattress suture. We have described what we believe to be a novel adaptation of the existing loop mattress suture to create the double loop mattress suture (Figure 1). We have also shown that the double loop mattress suture is stronger than the loop mattress and exerts a lower tension on the surrounding tissue.

In our experimental rig, we found that the double loop mattress suture could sustain loads that were 1.6× higher than the loop mattress and 3.2× higher than the simple suture conformation in cartilage. In delicate tissue, this effect was magnified: We found that the double loop mattress stitch is 3.5× stronger than the loop mattress and 6.9× stronger than the simple suture conformations in porcine skeletal muscle.

Interestingly, it was possible to calculate the stress–strain curves for cartilage samples according to suture conformation. We found that the type of stitch conformation employed heavily influences the mechanical behavior of the surrounding tissue into which it is inserted. The double loop mattress stitch made the surrounding tissue 1.5× stiffer than the loop mattress and 3.0× stiffer than the simple suture conformations. By comparison of Young's moduli when comparing the double loop and simple stitch conformations, this is the rough equivalent of uprating the stiffness of the cartilage from polypropylene, used in the manufacture of plastic bags, to pine wood along its grain. Increasing the stiffness of a tissue is only useful if it is not at the expense of the tissue's strength and ability to absorb energy. We looked at the modulus of resilience to investigate this and found that the double loop mattress stitch acts a bit like a spring in the tissue itself in terms of energy storage; we found that the cartilage could absorb 2.1× more energy with the double loop mattress than the loop mattress and 2.9× more energy than the simple suture conformations. This confirms that the tissue characteristics conferred by the double loop mattress stitch are conducive to wound healing.

To examine the effect of suture conformation on tissue tension, we created a very low-friction mechanical model (Figure 4A) with which we could test the mechanical advantage of each suture conformation. The mechanical advantage is the ratio of the effort required to appose a load: Higher mechanical advantages are found in systems where magnification of the effort is greater and are indicative of a lower exerted tissue tension, conducive to wound healing. The AMA for the double loop mattress stitch was 2.9, compared with 2.1 for the loop mattress and 1.5 for the simple suture conformations. The percentage effort required to move a fixed load is 66.6% for the simple, 71.4% for the mattress, 47.4% for the loop mattress, and 34.2% for the double loop mattress. These data expand on those presented by Gault and colleagues^4^ to show that the double loop mattress exerts a significant reduction in tissue tension for a fixed load in comparison with the loop mattress suture conformation. We believe that this reduction in tension can be explained through the increase in the number of times the suture strand crosses the wound (four vs. six between loop and double loop mattress stitches) and the formation of an additional loop to the previously described block and tackle arrangement possessed by the loop mattress stitch, enhancing the mechanical advantage of the double loop mattress suture through the introduction of an additional pulley.

We used SEM to look at the effect of suture conformation on tissue apposition and found that the double loop mattress and loop mattress stitches provided similar, significant reductions in horizontal apposition (wound gapping) compared with the simple conformation. Interestingly, we saw a significant improvement in vertical apposition (wound eversion) for the double loop mattress compared with both the loop mattress and simple stitch conformations. This difference was small but suggests that the double loop mattress stitch provides enhanced wound eversion with similar wound gapping when compared with the loop mattress.

We have validated these experimental results in vivo. Although the in vivo study was nonrandomized, the prospective data supports the laboratory findings that interrupted loop and double loop mattress sutures are superior to mattress. This innovative knotting technique would reduce the risk of wound dehiscence to almost zero. Based on these data, we recommend the use of loop mattress in most laparotomies and would favor the double loop mattress in clean laparotomy for tumor resection and in those patients undergoing secondary laparotomy.

The double loop mattress stitch is easily removed by cutting each of the loops and extracting the two threads from the wound. The nurses in our dressings clinics have been quickly trained to perform this task. We asked five experienced plastic surgery nurses to objectively rate the outcomes of the double loop mattress suture in the dressings clinic. We have shown that the double loop mattress stitch is as easy to remove as the loop mattress stitch and provides a comparable cosmetic outcome to the loop mattress stitch in vivo. In addition, we have seen no cases of loop-related wound edge necrosis as have been reported for the loop mattress stitch.^7^ We assume that this is because the tissue tension is reduced to a level that prevents microvascular compromise in this stitch conformation; however, should it occur over a larger case series, we would advocate the use of twisting to either one or both of the loops as has previously been described to prevent this.^7^

A double loop is the maximum achievable from a single suture strand in a mattress-type interrupted conformation, and only has one orientation. It is possible to create triple, quadruple, or even more loops by adapting the continuous mattress to incorporate a loop with the leading strand; however, it is practically very difficult to achieve an even distribution of force across the wound in this conformation as each loop effectively “locks” the trailing strand. Perpetual loops also raise the problem of excessive suture material in the wound. We did not see any cases of suture extrusion in our case series; however, it is important to use the double loop mattress stitch conformation judiciously for this reason. In addition to the case series presented, we have found the double loop mattress suture to be very useful in the fragile levator repair of cleft lip and palate deformity and to anchor key points of the keystone flap^13^ used in soft tissue repair. We have also found that a combination of absorbable and permanent double loop mattress stitches can be used effectively in very friable tissue to provide the maximum strength repair for the period of muscular healing and offer a permanence of effect. This principle can be extended to the repair of any skeletal muscle injury: The double loop mattress stitch conformation has a user-defined footprint that can be expanded in the repair of large muscles to provide a uniform and broad distribution of force that will prevent the “cheesewiring” which is often seen after skeletal muscle repair.

We have described the double loop mattress suture and demonstrated its superior strength in comparison with the loop mattress stitch. We have also shown that the double loop mattress exerts a lower tissue tension than the loop mattress and confers a stiffness and strength to tissues that will be of utility when repairing very friable, delicate tissue or as a dependable stay stitch in the repair of complex tissue defects. It is simple to tie, and we have used it in a variety of clinical scenarios where it has proved to be very useful as a reliable stitch that is very strong and exerts the minimum of tissue damage. In vivo, we found that the double loop mattress enhanced the edge eversion and decreased wound dehiscence compared with the mattress suture. We advocate its addition to the surgeon's toolbox for use in a wide variety of clinical situations where a high-strength, low-tension interrupted repair is required.

## Supporting Information

**Video S1**. Double loop mattress stitch construction.
